# Tokiinshi, a traditional Japanese medicine (Kampo), suppresses Panton-Valentine leukocidin production in the methicillin-resistant *Staphylococcus aureus* USA300 clone

**DOI:** 10.1371/journal.pone.0214470

**Published:** 2019-03-28

**Authors:** Yuka Maezawa, Hidemasa Nakaminami, Shunsuke Takadama, Minami Hayashi, Takeaki Wajima, Keisuke Nakase, Tetsuya Yamada, Hideaki Ikoshi, Norihisa Noguchi

**Affiliations:** 1 Department of Microbiology, School of Pharmacy, Tokyo University of Pharmacy and Life Sciences, Hachioji, Tokyo, Japan; 2 Department of Traditional Chinese Medicine, School of Pharmacy, Tokyo University of Pharmacy and Life Sciences, Hachioji, Tokyo, Japan; Indiana University School of Medicine-Northwest, UNITED STATES

## Abstract

It is necessary to develop agents other than antimicrobials for the treatment of *Staphylococcus aureus* infections to prevent the emergence of antimicrobial-resistant strains. Particularly, anti-virulence agents against the Panton-Valentine leukocidin (PVL)-positive methicillin-resistant *S*. *aureus* (MRSA), USA300 clone, is desired due to its high pathogenicity. Here, we investigated the potential anti-virulence effect of Tokiinshi, which is a traditional Japanese medicine (Kampo) used for skin diseases, against the USA300 clone. A growth inhibition assay showed that a conventional dose (20 mg/ml) of Tokiinshi has bactericidal effects against the clinical USA300 clones. Notably, the growth inhibition effects of Tokiinshi against *S*. *epidermidis* strains, which are the major constituents of the skin microbiome, was a bacteriostatic effect. The data suggested that Tokiinshi is unlikely to affect skin flora of *S*. *epidermidis*. Furthermore, PVL production and the expression of its gene were significantly suppressed in the USA300 clone by a lower concentration (5 mg/ml) of Tokiinshi. This did not affect the number of viable bacteria. Moreover, Tokiinshi significantly suppressed the expression of the *agrA* gene, which regulates PVL gene expression. For the first time, our findings strongly suggest that Tokiinshi has the potential to attenuate the virulence of the USA300 clone by suppressing PVL production via *agrA* gene suppression.

## Introduction

*Staphylococcus aureus* is one of the microbes that commonly inhabits human skin and nasal cavities. Conversely, the strains producing virulence factors cause various infectious diseases, such as skin infections, food poisoning, pneumonia, bacteremia, and toxic shock syndrome [1]. In particular, methicillin-resistant *S*. *aureus* (MRSA) is a causative agent of intractable infections. Panton-Valentine leukocidin (PVL) is one of the highly pathogenic toxins produced by *S*. *aureus* [2]. PVL is composed of two proteins: LukS-PV and LukF-PV; these two proteins associate on the target cell membranes to construct a six to eight mer holotoxin (membrane pore) and induce target cell necrosis [3, 4]. Thus, PVL is known to be associated with severe disorders, such as deep-seated skin infections, necrotizing fasciitis, and necrotizing pneumonia [5–7]. The USA300 clone, which is one of the PVL-positive MRSA lineages, shows high pathogenicity due to possession of an arginine catabolic mobile element (ACME) [8, 9]. Currently, the USA300 clone is widely disseminated in both community and healthcare settings and has become a pandemic clone [9]. Some other PVL-positive MRSA clones have also been identified globally [10]. One of them, the USA300-LV clone, is closely related to the USA300 clone and has spread rapidly through Latin American countries [11]. The Taiwan clone has mainly been found in Asian countries [7]. However, the USA300 clone is considered the most predominant and highly pathogenic PVL-positive MRSA.

Antimicrobial agents have been used for over half a century for treatment and prevention of bacterial infections. However, the development of antimicrobial-resistant bacteria, such as MRSA, has always been a problem. Even though new antimicrobial agents have been developed, bacteria rapidly acquire resistance against these new agents. Hence, the development of new and effective antibacterial agents is difficult. Additionally, antimicrobial agents often cannot recoup their considerable development costs for pharmaceutical companies, because they are usually used for only a short period. As a result, the development of new antimicrobial agents has been decreasing in recent years [12]. Furthermore, antibacterial agents affect our normal microbiome [13]. Therefore, an alternative approach, such as anti-virulence therapies that modulate bacterial toxin or virulence factors production [14], is necessary to resolve the above issues.

Together with *S*. *aureus*, *S*. *epidermis* is one constituent of the human skin microbiome. *S*. *epidermidis* produces glycerin from perspiration and sebum to maintain the skin barrier function [15]. In particular, *S*. *epidermidis* produces bacteriocin and antimicrobial peptides to prevent the growth of pathogenic bacteria on the skin [16, 17]. It has been reported that the reduction of the *S*. *epidermidis* population on the skin is associated with various disorders [18]. Thus, anti-virulence agents that exhibit less activity against *S*. *epidermidis* populations on the skin as compared to activity against MRSA that cause skin infections are desirable.

We have screened various herbal medicines to discover anti-virulence agents [19–21]. Tokiinshi is one of the traditional Japanese medicines (Kampo) used for skin diseases, such as eczema and atopic dermatitis [22]. It consists of 10 herbs, Angelica root, Rehmannia root, Tribulus fruit, Paeonia root, Cnidium rhizome, Saposhnikovia root, Polygonum root, Astragalus root, Schizonepeta spike, and Glycyrrhiza, and has been used in Japan since 1986. Some of the constituents have antimicrobial activity; however, the effects against *S*. *aureus* and *S*. *epidermidis* are unknown [23]. Here, we investigated the potential anti-virulence effect of Tokiinshi against clinical PVL-positive MRSA strains including the USA300 clone.

## Materials and methods

### Bacterial strains, growth conditions, and Kampo medicine preparation

The study protocols were approved by the Tokyo University of Pharmacy and Life Sciences Ethics Committee (13–13 and 16–12). Informed consent was not required from the patients and healthy individuals because the study did not involve clinical interactions with those subjects. We used four PVL-negative MRSA strains, nine PVL-positive MRSA strains (five USA300 clones, two USA300-LV clones, and two Taiwan clones), nine methicillin-susceptible *S*. *aureus* (MSSA) strains, and thirteen methicillin-susceptible *S*. *epidermidis* (MSSE) strains (Table 1) [7, 10]. MSSA ATCC29213 and methicillin-resistant *S*. *epidermidis* (MRSE) RP62A were used in a growth inhibition assay. All strains were cultivated in Mueller-Hinton agar (MHA, OXOID Ltd., Basingstoke, UK) or Mueller-Hinton broth (MHB, OXOID) at 35°C. Tokiinshi extract granules (Lot No. YH475) were purchased from Kotaro Pharmaceutical Co., Ltd. (Osaka, Japan). Contents of Tokiinshi were as follows; 4.0 g of Angelica root, 3.2 g of Rehmannia root, 2.4 g of Paeonia root, 2.4 g of Tribulus fruit, 2.4 g of Saposhnikovia root, 2.4 g of Cnidium rhizome, 2.0 g of Polygonum root, 1.6 g of Astragalus root, 1.6 g of Schizonepeta spike, and 0.8 g of Glycyrrhiza.

**Table 1:** Bacterial strains used in this study.

Species	Strain no.	Description [reference]
*S*. *aureus*	ATCC29213	Quality control strain for antimicrobial susceptibility testing
	15a, 17b, 30b, 45a, 56b, 64b, 69a, 70a, 72a	MSSA isolated from healthy skin [This study]
	TPS3119, TPS3160, TPS3332, TPS3355	PVL-negative clinical MRSA isolated from skin infections [7]
	TPS3472, TPS3517, TPS3993, TPS4353, TPS4655	USA300 clone (MRSA) isolated from skin infections [7]
	TPS3156, TPS3232	USA300-LV clone (MRSA) isolated from skin infections [7, 10]
	TPS4219, TPS4361	Taiwan clone (MRSA) isolated from skin infections [7]
*S*. *epidermidis*	RP62A (ATCC35984)	Laboratory stock MRSE strain
	2a, 11a, 19a, 24b, 34a, 45b, 46a, 52a, 53a, 62a, 64a, 75b, 78a	MSSE isolated from healthy skin [This study]

MSSA, methicillin-susceptible *Staphylococcus aureus*

MRSA, methicillin-resistant *Staphylococcus aureus*

MRSE, methicillin-resistant *Staphylococcus epidermidis*

MSSE, methicillin-susceptible *Staphylococcus epidermidis*

### Growth inhibition assay

Overnight cultures (4 × 10^3^ CFU/ml) of the tested strains were inoculated into MHB in the presence or absence of 20 mg/ml (a standard dose for oral use) of Tokiinshi and incubated with shaking for 6 h. The cultures were spread onto MHA at 0, 1, 2, 4, and 6 h of incubation. After 24 h, growth inhibition effects of Tokiinshi was determined by enumerating colony forming units (CFUs) on MHA. When MSSA and MRSE were co-cultured, the cultures were spread onto MHA in the presence or absence of 6 μg/ml oxacillin to distinguish MRSE. The results are shown as the mean ± standard error of the mean (SE) and log_10_ reduction values (LRVs) ± standard deviation (SD), which were derived from at least three independent experiments. The LRVs were calculated using the following formula: log_10_(CFU in the absence of Tokiinshi/CFU in the presence of Tokiinshi).

### PVL production inhibition assay

PVL production was measured using PVL-RPLA “Seiken” (DENKA SEIKEN Co., Ltd., Tokyo, Japan). Overnight cultures (4 × 10^3^ CFU/ml) of the tested strains were inoculated into MHB in the presence or absence of 5 mg/ml Tokiinshi and incubated with shaking for 20 h. The cultures were centrifuged at 3,000 × *g* for 20 min, and the supernatants were collected. The supernatants were serially diluted two-fold and added to 96-well microplates, and the PVL-sensitized latex was added and mixed thoroughly. After incubation at 25°C for 18 to 20 h, the agglutination titers were determined. The results were derived from at least two independent experiments.

### Preparation of bacterial RNA and real-time quantitative reverse-transcription polymerase chain reaction (qRT-PCR)

Total *S*. *aureus* RNA was isolated using a Blood / Cultured Cell Total RNA Mini Kit (Favorgen Biotech Corp., Ping-Tung, Taiwan). Overnight cultures (4 × 10^3^ CFU/ml) of the tested strains were inoculated into MHB in the presence or absence of Tokiinshi (1 to 5 mg/ml) and incubated with shaking for 10 h. Real-time qRT-PCR was performed using the cDNA prepared by ReverTra Ace (TOYOBO Co., Ltd., Osaka, Tokyo). Primers designed for the qRT-PCR assays are listed in S1 Table [24]. All samples were analyzed in triplicate, and expression levels normalized against *gmk* gene expression [25]. The results are shown as the mean ± SE, which were derived from at least three independent experiments.

### Statistical analysis

Differences in the number of viable bacteria (CFU/ml) between *S*. *aureus* ATCC29213 and *S*. *epidermidis* RP62A strains were compared using Welch’s *t*-test. Differences in the number of viable bacteria (CFU/ml) between clinical MRSA, MSSA, and MSSE strains were compared using Mann-Whitney U tests. The relative levels of PVL gene transcription were compared using Scheffé’s test following by a Kruskal-Wallis test. *P* values of less than 0.05 were considered statistically significant.

## Results

### Growth inhibition of *S*. *aureus* and *S*. *epidermidis*

**Fig 1:**
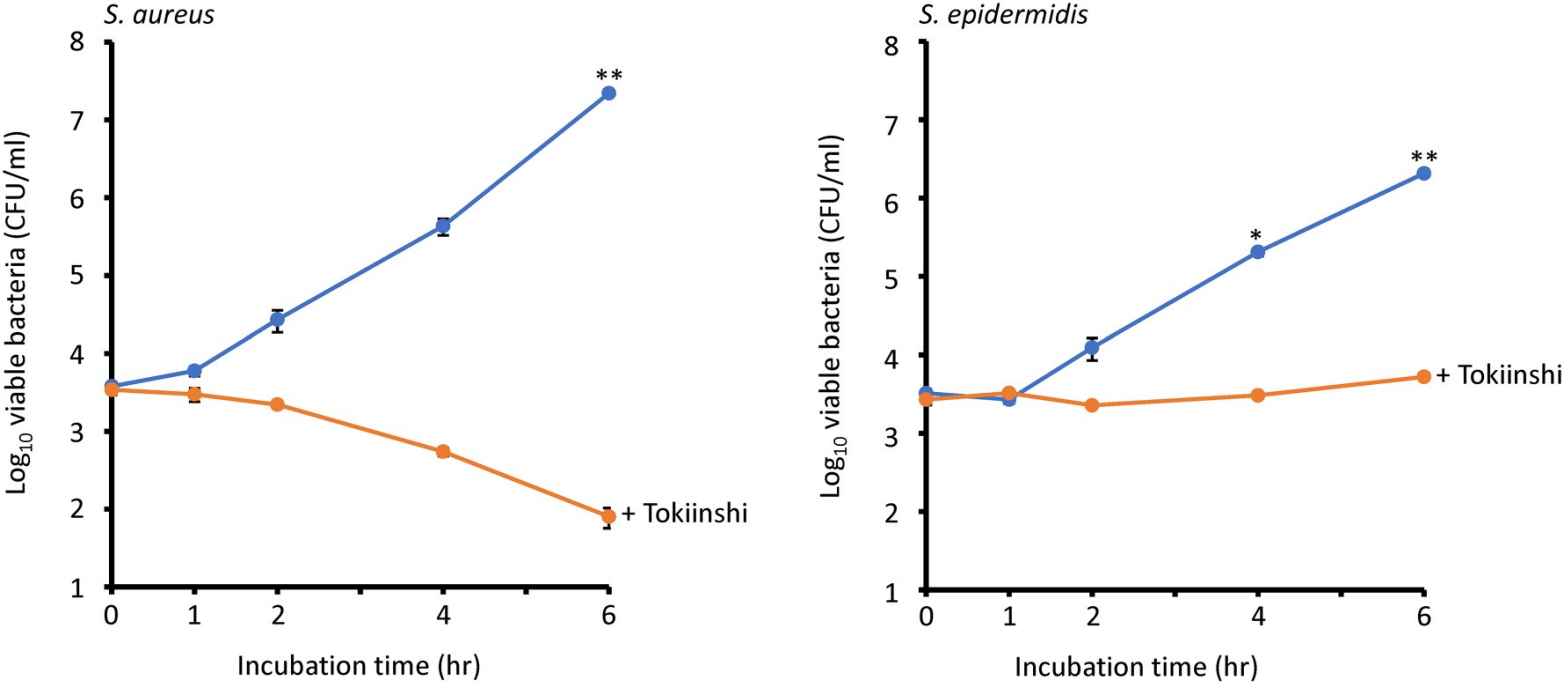
Growth inhibition effect of Tokiinshi (20 mg/ml) against *S*. *aureus* and *S*. *epidermidis*. *P < 0.05, **P < 0.01.

Treatment with 20 mg/ml Tokiinshi exhibited growth inhibition of both *S*. *aureus* ATCC29213 and *S*. *epidermidis* RP62A (Fig 1 and S2 Table). Specifically, the growth inhibition effect of Tokiinshi was greater against *S*. *aureus* than *S*. *epidermidis*. The LRV (log_10_ reduction value) at 6 h against *S*. *aureus* was more than 2-fold higher than that of *S*. *epidermidis*. To evaluate which component inhibits growth, we tested eight modified Tokiinshi formulas, each without one of the ten components of the original formula (S1 Fig). We found that each modified Tokiinshi formula was less effective at inhibiting the growth of *S*. *aureus* than the original Tokiinshi formula. In contrast, each modified formula was slightly more effective at inhibiting the growth of *S*. *epidermidis* than the original formula. Therefore, the data strongly suggest that all the herbal components of Tokiinshi are necessary for its bactericidal and bacteriostatic effects against *S*. *aureus* and *S*. *epidermidis*.

**Fig 2:**
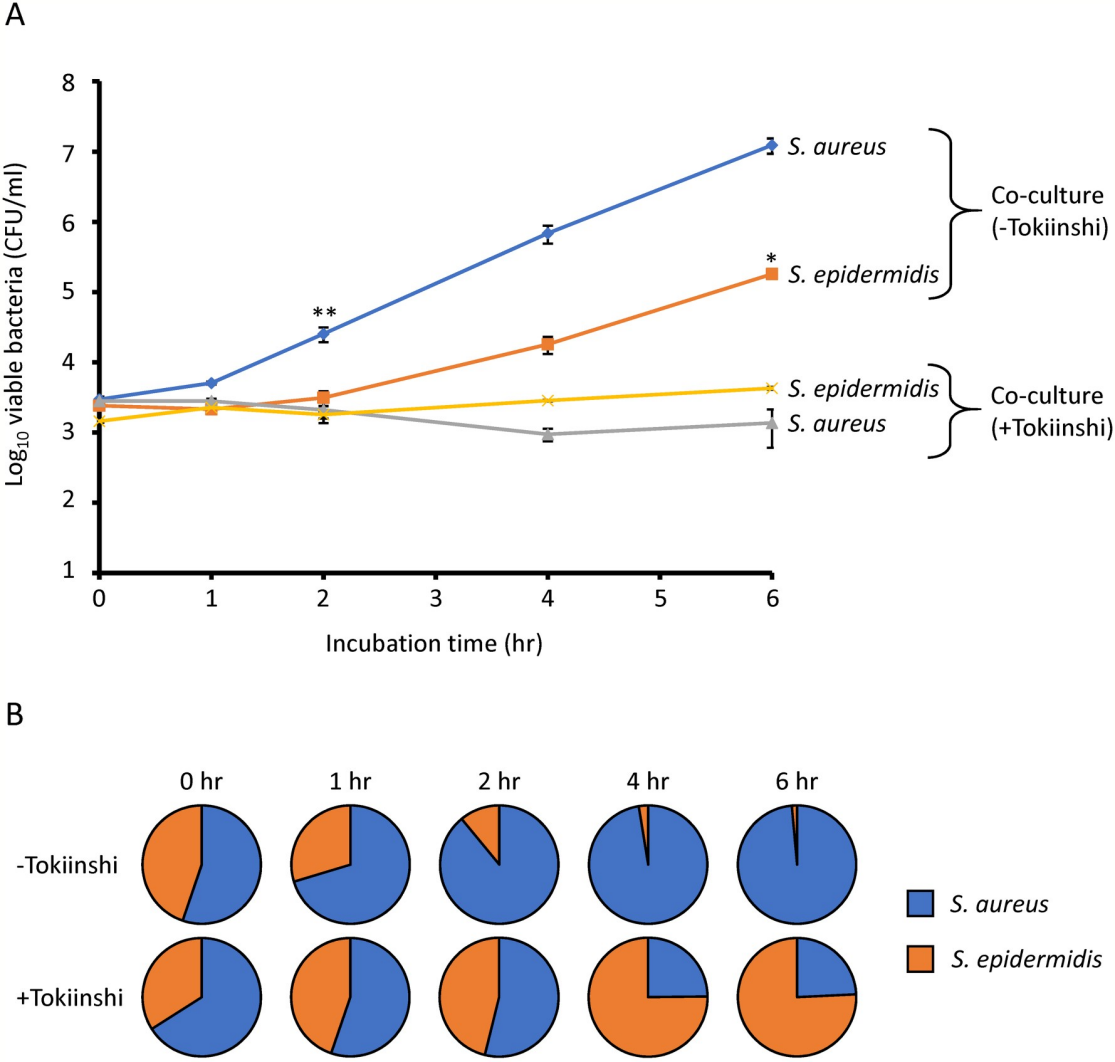
Growth inhibition effect of Tokiinshi (20 mg/ml) against co-culture of *S*. *aureus* and *S*. *epidermidis*. A, Growth curve of *S*. *aureus* and *S*. *epidermidis* in the co-culture. B, Survival ratio of *S*. *aureus* and *S*. *epidermidis* in the co-culture. *P < 0.05, in the presence of Tokiinshi, **P < 0.01, in the presence of Tokiinshi.

In most cases of skin infections caused by *S*. *aureus*, it is predicted that *S*. *epidermidis* exists in the same region. Hence, we investigated the growth inhibition effects of Tokiinshi against co-cultured *S*. *aureus* and *S*. *epidermidis* (Fig 2). The survival ratios of *S*. *epidermidis* in the absence of Tokiinshi were 29.7% (1 h), 11.0% (2 h), 2.6% (4 h), and 1.4% (6 h). By contrast, the ratios spiked in the presence of 20 mg/ml Tokiinshi as follows; 44.7% (1 h), 46.1% (2 h), 75.2% (4 h), and 75.8% (6 h). Therefore, the data showed that treatment with 20 mg/ml Tokiinshi exhibited bactericidal effect against *S*. *aureus*, whereas the effect was bacteriostatic against *S*. *epidermidis*. The growth curves for *S*. *aureus* and *S*. *epidermidis* in the presence and absence of 5 mg/ml Tokiinshi revealed only a slight difference between control and Tokiinshi in the number of viable bacteria of both strains after exposure for 20 h (S2 Fig).

### Growth inhibition effect against clinical *S*. *aureus* strains

**Fig 3:**
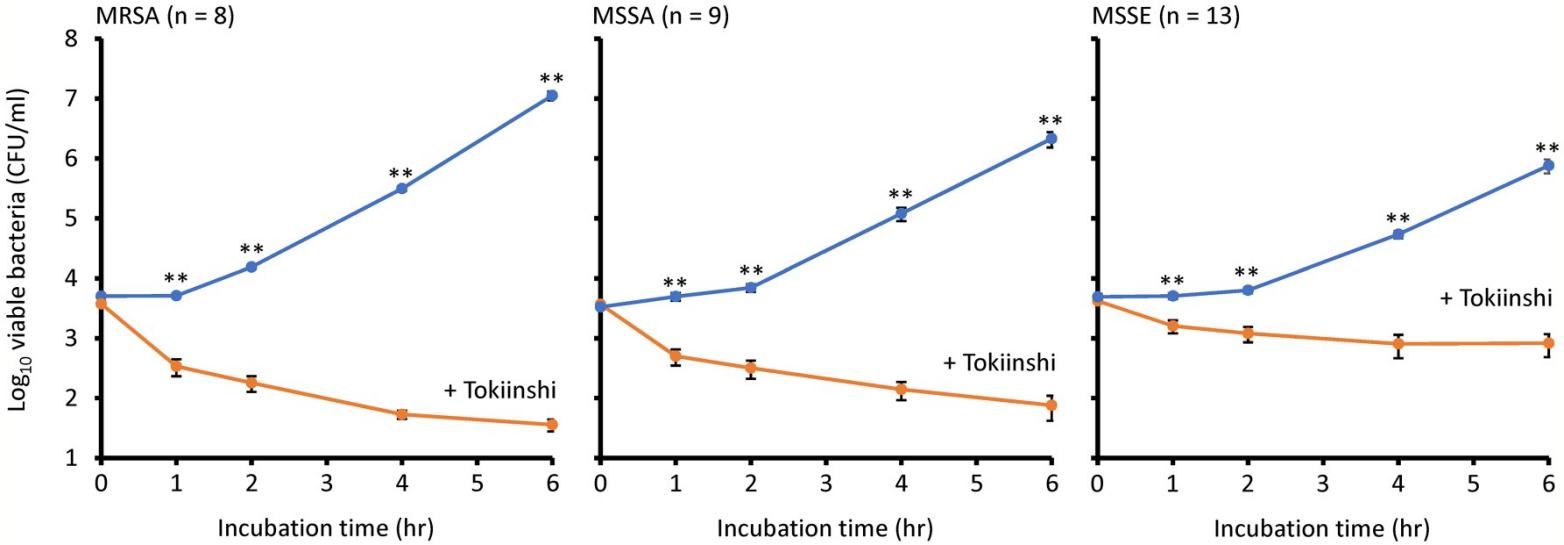
Growth inhibition effect of Tokiinshi (20 mg/ml) against clinical MRSA, MSSA, and MSSE strains. MRSA, methicillin-resistant *Staphylococcus aureus*. MSSA, methicillin-susceptible *Staphylococcus aureus*. MSSE, methicillin-susceptible *Staphylococcus epidermidis*. **P < 0.01.

To evaluate the bactericidal effect of Tokiinshi against clinical staphylococcal isolates, we determined the growth inhibition effect of 20 mg/ml Tokiinshi against eight MRSA isolates including four PVL-positive strains (two USA300-LV clones, one USA300 clone, and one Taiwan clone) derived from patients with skin infections, and nine MSSA and 13 MSSE isolates derived from healthy individuals (Table 1, Fig 3, and S3 Table). The LRVs at 1 h against MRSA (1.37 ± 0.42) and MSSA (1.18 ± 0.59) were 2-fold higher than that of *S*. *epidermidis* (0.68 ± 0.48). Similar results were also observed at every time point. Therefore, 20 mg/ml Tokiinshi also exerts a bactericidal effect against clinical MRSA isolates including PVL-positive strains, and a bacteriostatic effect against *S*. *epidermidis* isolates from the healthy skin microbiome.

### Suppression of PVL production and expression

**Fig 4:**
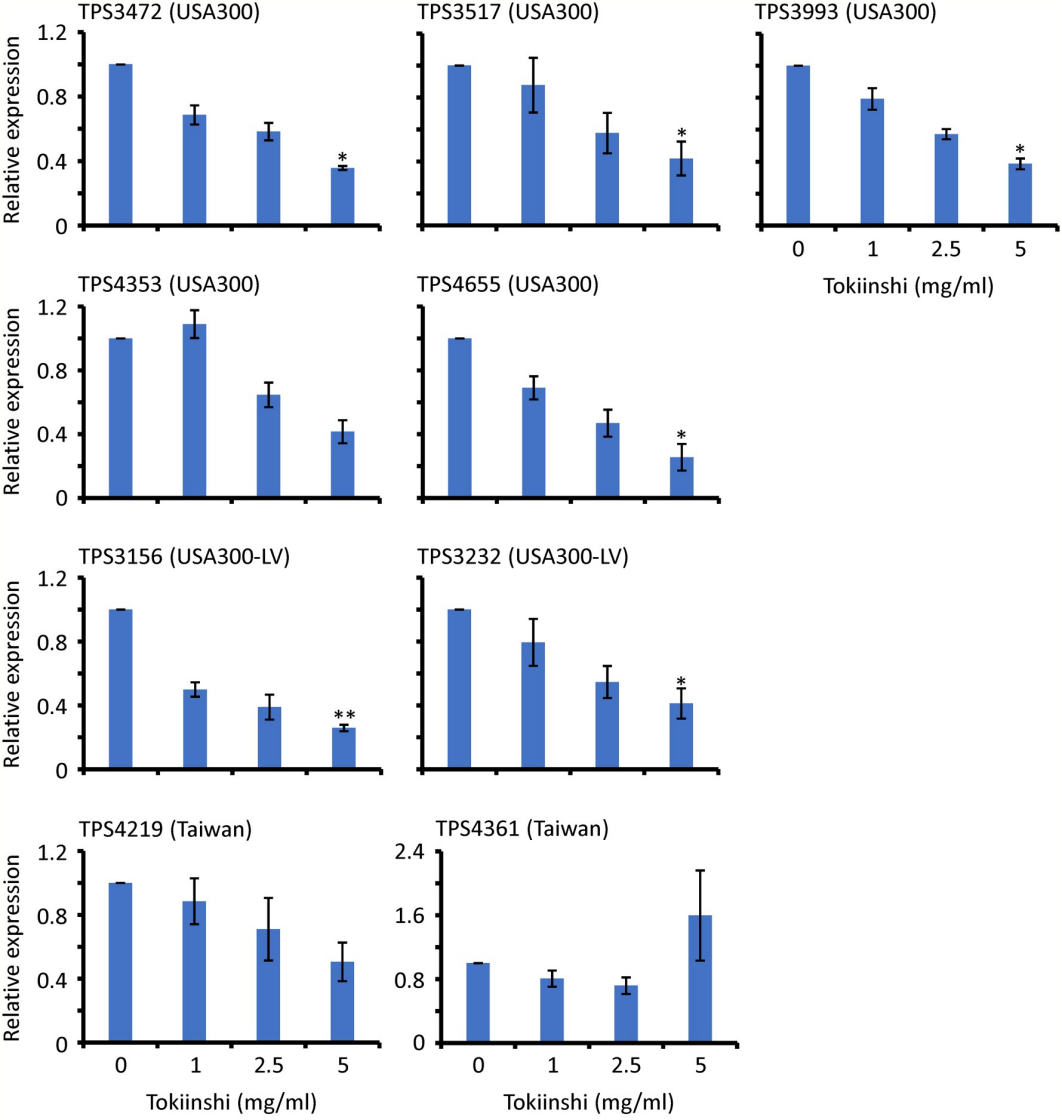
PVL gene expression in the presence and absence of Tokiinshi. *P < 0.05 and **P < 0.01 vs. 0 mg/ml.

To determine whether Tokiinshi has a potential of anti-virulence effect, PVL production was determined in the presence and absence of 5 mg/ml Tokiinshi (Table 2). We confirmed that 5 mg/ml Tokiinshi did not affect viable bacterial counts after 20 h exposure. Tokiinshi suppressed PVL production 4- to 16-fold. PVL production varies depending on the genotypes of the strains. Specifically, PVL production in the Taiwan clone was 2- to 16-fold lower than that of the other clones. However, suppression was not observed with ≤2.5 mg/ml Tokiinshi treatment. Next, to investigate whether Tokiinshi affects PVL gene expression, real-time qRT-PCR was conducted (Fig 4). The PVL gene expressions of the tested MRSA strains excluding TPS4361 (Taiwan clone) were suppressed in a concentration-dependent manner by Tokiinshi treatment. Compared with the Taiwan clone, the suppression levels were higher in the USA300 and the USA300-LV clones. The PVL gene suppression by Tokiinshi could not be found in one of the Taiwan clones, TPS4361. Therefore, the PVL suppression by Tokiinshi was more effective against the high PVL-producing strains, the USA300 and USA300-LV clones.

**Table 2:** PVL production in the presence and absence of Tokiinshi (5 mg/ml).

Clone	Strain no.	PVL production (titer) [CFU/ml]*		Relative production [Tokiinshi (-)/(+)]
		Tokiinshi (-)	Tokiinshi (+)	
USA300	TPS3472	×128 [6.8 ± 1.7 × 10^9^]	×16 [8.2 ± 1.8 × 10^9^]	8
	TPS3517	×64 [6.6 ± 1.6 × 10^9^]	×8 [4.4 ± 0.01 × 10^9^]	8
	TPS3993	×128 [7.4 ± 1.2 × 10^9^]	×16 [1.2 ± 0.2 × 10^10^]	8
	TPS4353	×128 [8.2 ± 1.3 × 10^9^]	×16 [9.4 ± 1.8 × 10^9^]	8
	TPS4655	×128 [6.2 ± 0.3 × 10^9^]	×32 [1.7 ± 0.8 × 10^10^]	4
USA300-LV	TPS3156	×128 [5.4 ± 0.4 × 10^9^]	×8 [5.3 ± 0.8 × 10^9^]	16
	TPS3232	×64 [4.8 ± 0.3 × 10^9^]	×4 [5.5 ± 1.4 × 10^9^]	16
Taiwan clone	TPS4219	×32 [5.6 ± 1.7 × 10^9^]	×8 [4.9 ± 0.01 × 10^9^]	4
	TPS4361	×8 [4.7 ± 0.7 × 10^9^]	×1 [4.0 ± 2.4 × 10^9^]	8

*CFU/ml data are shown as mean ± standard error of mean (SE).

### Suppression of *agrA* and *hla* genes expression

**Fig 5:**
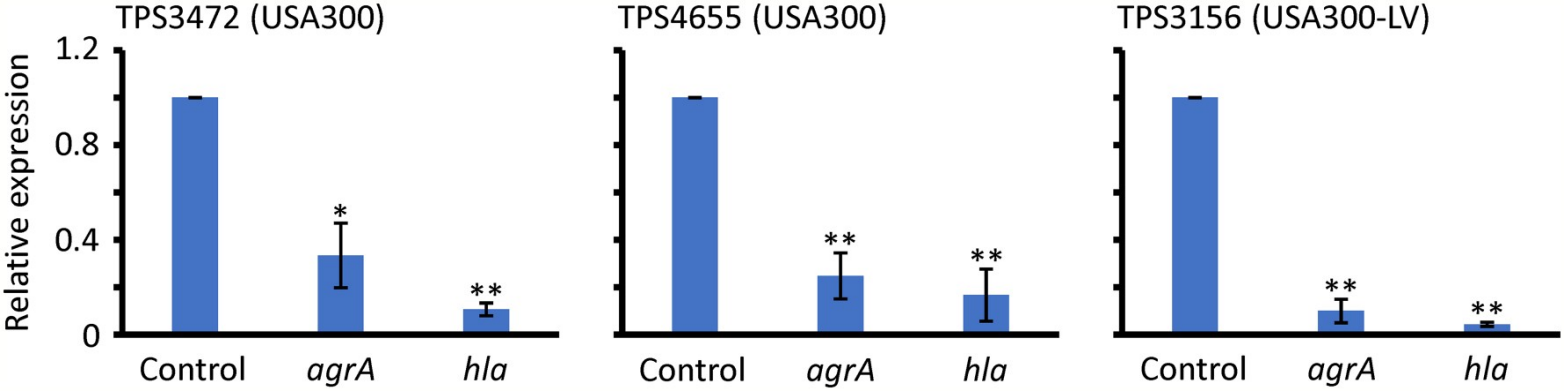
*agrA* and *hla* genes expression in the presence and absence of Tokiinshi (2.5 mg/ml). *P < 0.05 and **P < 0.01 vs. control.

PVL gene expression is regulated by an accessory gene regulator (*agr*) [26]. The *agrA* gene also regulates the production of Hla, which is an α-hemolysin and an essential virulence factor of *S*. *aureus* [27]. Therefore, we used real-time qRT-PCR to determine the expression levels of *agrA* and *hla* genes in *S*. *aureus* exposed to 2.5 mg/ml Tokiinshi (Fig 5). Tokiinshi significantly suppressed the expression of both *agrA* and *hla*. The data suggest that the reduced production and expression of PVL were caused by the suppression of the *agrA* gene by Tokiinshi.

## Discussion

Our data showed that a conventional concentration (20 mg/ml) of Tokiinshi, which is a Kampo medicine used for skin diseases, has bactericidal and bacteriostatic activities against *S*. *aureus* and *S*. *epidermidis*, respectively. The growth rates of *S*. *aureus* strains were faster than those of *S*. *epidermidis* strains (Figs 2 and 3). These results suggested that Tokiinshi mainly acts against actively growing bacteria. Tokiinshi may exert such growth inhibition against a wide range of *S*. *aureus* and *S*. *epidermidis* strains, because the same effects were also found in clinical isolates from patients with skin infections and healthy individuals. Furthermore, Tokiinshi increased the survival ratio of *S*. *epidermidis* when co-cultured with *S*. *aureus*. The bactericidal effect of Tokiinshi on *S*. *aureus* decreased slightly following co-culture with *S*. *epidermidis*. Although the reason for this is unclear, we hypothesize that some active components in Tokiinshi might be degraded by *S*. *epidermidis* enzymes. The skin microbiome is known to affect immune function of the host, e.g., atopic dermatitis [28]. *S*. *aureus* is a highly pathogenic bacterium due to its ability to produce various toxins [1], while *S*. *epidermidis* is an indigenous bacterium that helps to form normal skin flora and contributes to host immune function [16, 17]. Therefore, we considered that Tokiinshi is a useful agent for maintaining homeostasis of the skin due to its specific bactericidal activity against *S*. *aureus*. However, the growth inhibition effect of Tokiinshi decreased with reduced concentration. Bacteriostatic activity was retained in 10 mg/ml of Tokiinshi treatment, but 5 mg/ml treatment could not inhibit bacterial growth. We do not have any idea of the concentration of Tokiinshi that will reach the skin after oral administration. Hence, we recommend that Tokiinshi be applied directly to the skin as a topical agent.

Notably, Tokiinshi suppressed PVL production, which varies depending on the strain genotypes. Particularly, the USA300 and USA300-LV clones, which are much more highly pathogenic strains than Taiwan clone [7], produced higher levels of PVL than that of the Taiwan clone. Tokiinshi could suppress the PVL production of the USA300 and USA300-LV clones to the same level as that of the Taiwan clone. Additionally, we demonstrated that PVL suppression was caused by affecting gene expression. Moreover, Tokiinshi significantly suppressed the expression of the *agrA* and *hla* genes. The reduced expression of *agrA* suppressed *hla*. Therefore, the data strongly suggest that Tokiinshi attenuates the virulence of highly pathogenic PVL-positive MRSA by suppressing PVL production via *agrA* gene suppression.

Dumitrescu et al. reported that some antimicrobial agents used for *S*. *aureus* infections suppress PVL production [29]. However, antimicrobial agents inhibit not only pathogenic bacteria but also the host microbiome. By contrast, Tokiinshi could suppress PVL production without bacterial growth inhibition. Tokiinshi has been used for treating skin diseases in Japan since 1986, and no severe side effects have been reported. Therefore, Tokiinshi has a potential to become an anti-virulence agent against severe skin infections caused by the USA300 clone. We recommend that Tokiinshi be used as an adjunct agent for antimicrobial therapy. Further study is necessary to evaluate the synergistic effects of Tokiinshi and antimicrobial agents.

The present study has some limitations. First, we did not determine which components of Tokiinshi are essential for the suppression of PVL production. However, the pharmacological activity of Kampo medicines generally depends on all their components. Hence, we predict that the components cannot suppress PVL production individually. Second, we did not assess the *in vivo* anti-virulence efficacy of Tokiinshi. Further experiments, such as animal experiments and cytotoxicity assays, are necessary to validate our data. Additionally, clinical studies are necessary to evaluate the availability of Tokiinshi against skin infections caused by PVL-positive MRSA.

## Conclusions

Our findings strongly suggest, for the first time, that Tokiinshi has the potential to become an anti-virulence agent against severe skin infections caused by the USA300 clone. Clinical studies are necessary to evaluate the activity of Tokiinshi against skin infections caused by PVL-positive MRSA.

## Supporting information

S1 Fig Growth inhibition effect of eight herbal drugs without one of the components of Tokiinshi against *S*. *aureus* and *S*. *epidermidis*. (TIFF)

S2 Fig Growth inhibition effect of Tokiinshi (5 mg/ml) against *S*. *aureus* and *S*. *epidermidis*. (TIFF)

S1 Table Primers used in this study. (DOCX)

S2 Table Log_10_ reduction values of *Staphylococcus aureus* and *Staphylococcus epidermidis* by Tokiinshi (20 mg/ml). (DOCX)

S3 Table Log_10_ reduction values of methicillin-resistant *Staphylococcus aureus* (a), methicillin-susceptible *Staphylococcus aureus* (b), and methicillin-susceptible *Staphylococcus epidermidis* (c) by Tokiinshi (20 mg/ml). (DOCX)

S1 Dataset Raw data for all figures and tables in this manuscript. (XLSX)

## References

[1] Lowy FD. *Staphylococcus aureus* infections. N Engl J Med. 1998;339(8):520–32. 10.1056/NEJM199808203390806 9709046

[2] Yamamoto T, Nishiyama A, Takano T, Yabe S, Higuchi W, Razvina O, et al Community-acquired methicillin-resistant *Staphylococcus aureus*: community transmission, pathogenesis, and drug resistance. J Infect Chemother. 2010;16(4):225–54. 10.1007/s10156-010-0045-9 20336341 PMC7088255

[3] Kaneko J, Kamio Y. Bacterial two-component and hetero-heptameric pore-forming cytolytic toxins: structures, pore-forming mechanism, and organization of the genes. Biosci Biotechnol Biochem. 2004;68(5):981–1003. 10.1271/bbb.68.981 15170101

[4] Genestier AL, Michallet MC, Prevost G, Bellot G, Chalabreysse L, Peyrol S, et al *Staphylococcus aureus* Panton-Valentine leukocidin directly targets mitochondria and induces Bax-independent apoptosis of human neutrophils. J Clin Invest. 2005;115(11):3117–27. 10.1172/JCI22684 16276417 PMC1265849

[5] Gillet Y, Issartel B, Vanhems P, Fournet JC, Lina G, Bes M, et al Association between *Staphylococcus aureus* strains carrying gene for Panton-Valentine leukocidin and highly lethal necrotising pneumonia in young immunocompetent patients. Lancet. 2002;359(9308):753–9. 10.1016/S0140-6736(02)07877-7 11888586

[6] Miller LG, Perdreau-Remington F, Rieg G, Mehdi S, Perlroth J, Bayer AS, et al Necrotizing fasciitis caused by community-associated methicillin-resistant *Staphylococcus aureus* in Los Angeles. N Engl J Med. 2005;352(14):1445–53. 10.1056/NEJMoa042683 15814880

[7] Takadama S, Nakaminami H, Aoki S, Akashi M, Wajima T, Ikeda M, et al Prevalence of skin infections caused by Panton-Valentine leukocidin-positive methicillin-resistant *Staphylococcus aureus* in Japan, particularly in Ishigaki, Okinawa. J Infect Chemother. 2017;23(11):800–3. 10.1016/j.jiac.2017.04.016 28552322

[8] Tenover FC, Goering RV. Methicillin-resistant *Staphylococcus aureus* strain USA300: origin and epidemiology. J Antimicrob Chemother. 2009;64(3):441–6. 10.1093/jac/dkp241 19608582

[9] Nimmo GR. USA300 abroad: global spread of a virulent strain of community-associated methicillin-resistant *Staphylococcus aureus*. Clin Microbiol Infect. 2012;18(8):725–34. 10.1111/j.1469-0691.2012.03822.x 22448902

[10] Takadama S, Nakaminami H, Sato A, Shoshi M, Fujii T, Noguchi N. Dissemination of Panton-Valentine leukocidin-positive methicillin-resistant *Staphylococcus aureus* USA300 clone in multiple hospitals in Tokyo, Japan. Clin Microbiol Infect. 2018 10.1016/j.cmi.2018.02.012 29454850

[11] Planet PJ, Diaz L, Kolokotronis SO, Narechania A, Reyes J, Xing G, et al Parallel epidemics of community-associated methicillin-resistant *Staphylococcus aureus* USA300 infection in North and South America. J Infect Dis. 2015;212(12):1874–82. 10.1093/infdis/jiv320 26048971 PMC4655856

[12] Infectious Diseases Society of A, Spellberg B, Blaser M, Guidos RJ, Boucher HW, Bradley JS, et al Combating antimicrobial resistance: policy recommendations to save lives. Clin Infect Dis. 2011;52 Suppl 5:S397–428. 10.1093/cid/cir153 21474585 PMC3738230

[13] Morgun A, Dzutsev A, Dong X, Greer RL, Sexton DJ, Ravel J, et al Uncovering effects of antibiotics on the host and microbiota using transkingdom gene networks. Gut. 2015;64(11):1732–43. 10.1136/gutjnl-2014-308820 25614621 PMC5166700

[14] Kong C, Neoh HM, Nathan S. Targeting *Staphylococcus aureus* Toxins: A Potential form of Anti-Virulence Therapy. Toxins (Basel). 2016;8(3). 10.3390/toxins8030072 26999200 PMC4810217

[15] Wang Y, Kuo S, Shu M, Yu J, Huang S, Dai A, et al *Staphylococcus epidermidis* in the human skin microbiome mediates fermentation to inhibit the growth of *Propionibacterium acnes*: implications of probiotics in acne vulgaris. Appl Microbiol Biotechnol. 2014;98(1):411–24. 10.1007/s00253-013-5394-8 24265031 PMC3888247

[16] Lai Y, Cogen AL, Radek KA, Park HJ, Macleod DT, Leichtle A, et al Activation of TLR2 by a small molecule produced by *Staphylococcus epidermidis* increases antimicrobial defense against bacterial skin infections. J Invest Dermatol. 2010;130(9):2211–21. 10.1038/jid.2010.123 20463690 PMC2922455

[17] Christensen GJ, Bruggemann H. Bacterial skin commensals and their role as host guardians. Benef Microbes. 2014;5(2):201–15. 10.3920/BM2012.0062 24322878

[18] Laborel-Preneron E, Bianchi P, Boralevi F, Lehours P, Fraysse F, Morice-Picard F, et al Effects of the *Staphylococcus aureus* and *Staphylococcus epidermidis* Secretomes Isolated from the Skin Microbiota of Atopic Children on CD4+ T Cell Activation. PLoS One. 2015;10(10):e0141067 10.1371/journal.pone.0141067 26510097 PMC4624846

[19] Yamada T, Wajima T, Nakaminami H, Kobayashi K, Ikoshi H, Noguchi N. The modified Gingyo-san, a Chinese herbal medicine, has direct antibacterial effects against respiratory pathogens. BMC Complement Altern Med. 2016;16(1):463 10.1186/s12906-016-1431-3 27842538 PMC5109643

[20] Wajima T, Anzai Y, Yamada T, Ikoshi H, Noguchi N. Oldenlandia diffusa Extract Inhibits Biofilm Formation by *Haemophilus influenzae* Clinical Isolates. PLoS One. 2016;11(11):e0167335 10.1371/journal.pone.0167335 27902758 PMC5130263

[21] Wajima T, Kinugawa R, Yamada T, Ikoshi H, Noguchi N. Panax Notoginseng Extract Possesses Significant Antibacterial Activity against Pathogenic Streptococci. Pharmacology. 2019;103(5–6):221–7. 10.1159/000496830 30690443

[22] Nose M, Sakushima J, Harada D, Ogihara Y. Comparison of immunopharmacological actions of 8 kinds of kampo-hozais clinically used in atopic dermatitis on delayed-type hypersensitivity in mice. Biol Pharm Bull. 1999;22(1):48–54. 9989661 10.1248/bpb.22.48

[23] Wang L, Yang R, Yuan B, Liu Y, Liu C. The antiviral and antimicrobial activities of licorice, a widely-used Chinese herb. Acta Pharm Sin B. 2015;5(4):310–5. 10.1016/j.apsb.2015.05.005 26579460 PMC4629407

[24] Kaito C, Saito Y, Nagano G, Ikuo M, Omae Y, Hanada Y, et al Transcription and translation products of the cytolysin gene psm-mec on the mobile genetic element SCC*mec* regulate *Staphylococcus aureus* virulence. PLoS Pathog. 2011;7(2):e1001267 10.1371/journal.ppat.1001267 21304931 PMC3033363

[25] Nakaminami H, Chen C, Truong-Bolduc QC, Kim ES, Wang Y, Hooper DC. Efflux Transporter of Siderophore Staphyloferrin A in *Staphylococcus aureus* Contributes to Bacterial Fitness in Abscesses and Epithelial Cells. Infect Immun. 2017;85(8). 10.1128/IAI.00358-17 28559406 PMC5520428

[26] Villaruz AE, Bubeck Wardenburg J, Khan BA, Whitney AR, Sturdevant DE, Gardner DJ, et al A point mutation in the agr locus rather than expression of the Panton-Valentine leukocidin caused previously reported phenotypes in *Staphylococcus aureus* pneumonia and gene regulation. J Infect Dis. 2009;200(5):724–34. 10.1086/604728 19604047 PMC2777534

[27] Khodaverdian V, Pesho M, Truitt B, Bollinger L, Patel P, Nithianantham S, et al Discovery of antivirulence agents against methicillin-resistant *Staphylococcus aureus*. Antimicrob Agents Chemother. 2013;57(8):3645–52. 10.1128/AAC.00269-13 23689713 PMC3719762

[28] Sanford JA, Gallo RL. Functions of the skin microbiota in health and disease. Semin Immunol. 2013;25(5):370–7. 10.1016/j.smim.2013.09.005 24268438 PMC4219649

[29] Dumitrescu O, Badiou C, Bes M, Reverdy ME, Vandenesch F, Etienne J, et al Effect of antibiotics, alone and in combination, on Panton-Valentine leukocidin production by a *Staphylococcus aureus* reference strain. Clin Microbiol Infect. 2008;14(4):384–8. 10.1111/j.1469-0691.2007.01947.x 18261123

