# Supplementary figures and images for: Tokiinshi, a traditional Japanese medicine (Kampo), suppresses Panton-Valentine leukocidin production in the methicillin-resistant Staphylococcus aureus USA300 clone
Source: PLoS One. 2019 Mar 28;14(3):e0214470. doi: 10.1371/journal.pone.0214470 (PMC6438529; doi:10.1371/journal.pone.0214470)

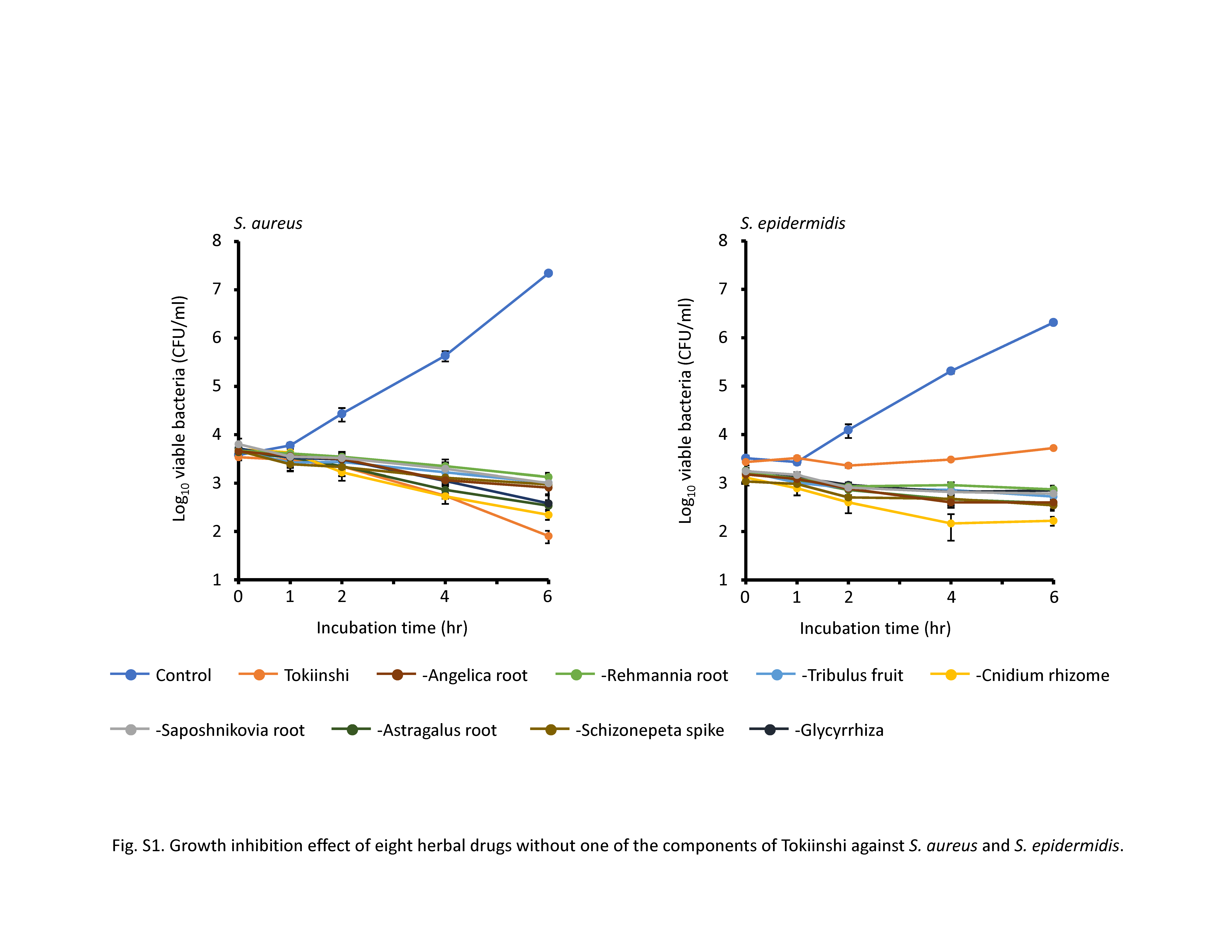

Supplement: S1 Fig — (TIFF) [file pone.0214470.s001.tiff]

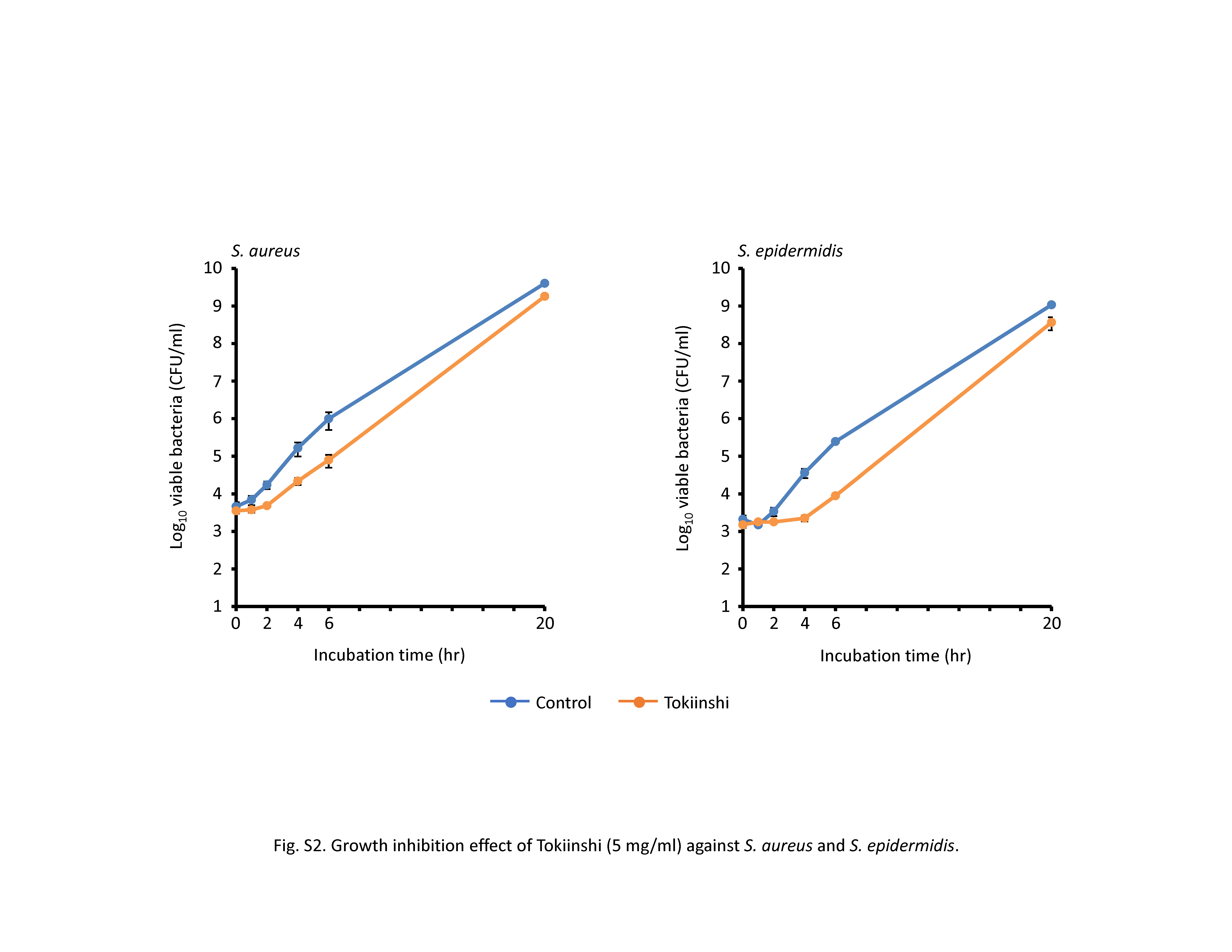

Supplement: S2 Fig — (TIFF) [file pone.0214470.s002.tiff]
