# Supplementary material for: Tokiinshi, a traditional Japanese medicine (Kampo), suppresses Panton-Valentine leukocidin production in the methicillin-resistant Staphylococcus aureus USA300 clone
Source: PLoS One. 2019 Mar 28;14(3):e0214470. doi: 10.1371/journal.pone.0214470 (PMC6438529; doi:10.1371/journal.pone.0214470)
Supplement: S1 Table — (DOCX) [file pone.0214470.s003.docx]

S1 Table. Primers used in this study

Target gene Direction Sequence Product size (bp) Reference

*pvl* Forward CACAGTTAAATATGAAGTGAACTGGA 104 This study
 Reverse TGCAATTGATGTAACAACTGATG
 *gmk* Forward TGCTGAATATGTAGGCAACTATTATG 114 24
 Reverse CTAACTTGCTTTGCACCTTCTACT
 *agrA* Forward GCCCTCGCAACTGATAATCCT 147 23
 Reverse ACCAACTGGGTCATGCTTACG
 *hla* Forward GGTGCAAATGTTTCGATTGG 277 23
 Reverse CGAAGTCTGGTGAAAACCCTGA
