# Supplementary material for: Tokiinshi, a traditional Japanese medicine (Kampo), suppresses Panton-Valentine leukocidin production in the methicillin-resistant Staphylococcus aureus USA300 clone
Source: PLoS One. 2019 Mar 28;14(3):e0214470. doi: 10.1371/journal.pone.0214470 (PMC6438529; doi:10.1371/journal.pone.0214470)
Supplement: S2 Table — (DOCX) [file pone.0214470.s004.docx]

S2 Table. Log_10_ reduction values of *Staphylococcus aureus* and *Staphylococcus epidermidis* by Tokiinshi (20 mg/ml)

Time (hr) Log reduction value ± SD

*S. aureus* *S. epidermidis*
 1 0.31 ± 0.18 -0.09 ± 0.12
 2 1.05 ± 0.21 0.70 ± 0.22
 4 2.88 ± 0.19 1.82 ± 0.10
 6 5.48 ± 0.17 2.59 ± 0.05
