# Supplementary material for: Tokiinshi, a traditional Japanese medicine (Kampo), suppresses Panton-Valentine leukocidin production in the methicillin-resistant Staphylococcus aureus USA300 clone
Source: PLoS One. 2019 Mar 28;14(3):e0214470. doi: 10.1371/journal.pone.0214470 (PMC6438529; doi:10.1371/journal.pone.0214470)
Supplement: S3 Table — (DOCX) [file pone.0214470.s005.docx]

S3 Tables. Log10 reduction values of methicillin-resistant *Staphylococcus aureus* (a), methicillin-susceptible *Staphylococcus aureus* (b), and methicillin-susceptible *Staphylococcus epidermidis* (c) by Tokiinshi (20 mg/ml).

(a)

Time (hr) Log reduction value ± SD

TPS3119 TPS3160 TPS3332 TPS3355 TPS3156 TPS3232 TPS3517 TPS4219 Average
 1 1.88 ± 0.38 1.81 ± 0.07 1.36 ± 0.24 1.76 ± 0.27 0.87 ± 0.13 0.91 ± 0.13 0.84 ± 0.24 1.56 ± 0.32 1.37 ± 0.42
 2 2.46 ± 0.18 2.55 ± 0.05 1.98 ± 0.13 2.33 ± 0.16 1.74 ± 0.15 1.83 ± 0.23 1.50 ± 0.22 2.33 ± 0.08 2.09 ± 0.36
 4 3.74 ± 0.18 3.83 ± 0.16 3.93 ± 0.12 4.03 ± 0.23 3.62 ± 0.27 3.80 ± 0.47 3.58 ± 0.29 3.86 ± 0.23 3.80 ± 0.14
 6 5.64 ± 0.26 5.73 ± 0.29 5.37 ± 0.40 5.65 ± 0.48 4.81 ± 0.54 5.44 ± 0.38 4.88 ± 0.38 5.79 ± 0.37 5.41 ± 0.35

(b)

Time (hr) Log reduction value ± SD

15a 17b 30b 45a 56b 64b 69a 70a 72a Average
 1 1.08 ± 0.08 1.95 ± 0.15 0.56 ± 0.12 1.22 ± 0.25 0.39 ± 0.14 0.53 ± 0.13 2.18 ± 0.16 1.23 ± 0.07 1.52 ± 0.09 1.18 ± 0.59
 2 1.44 ± 0.05 2.32 ± 0.05 0.95 ± 0.01 1.66 ± 0.05 0.73 ± 0.14 0.68 ± 0.12 2.37 ± 0.18 1.70 ± 0.10 1.92 ± 0.17 1.53 ± 0.60
 4 2.80 ± 0.16 3.54 ± 0.09 2.88 ± 0.08 3.78 ± 0.22 2.33 ± 0.38 2.04 ± 0.07 4.05 ± 0.23 3.01 ± 0.16 3.09 ± 0.37 3.06 ± 0.62
 6 4.65 ± 0.12 4.97 ± 0.25 4.64 ± 0.19 5.37 ± 0.18 3.93 ± 0.40 3.19 ± 0.22 5.09 ± 0.63 4.09 ± 0.21 4.36 ± 0.18 4.48 ± 0.63

(c)

Time (hr) Log reduction value ± SD

2a 11a 19a 24b 34a 45b 46a 52a 53a 62a 64a 75b 78a Average
 1 -0.08 ± 0.11 1.18 ± 0.16 0.29 ± 0.06 0.42 ± 0.12 0.54 ± 0.18 0.38 ± 0.18 0.72 ± 0.16 1.36 ± 0.37 0.90 ± 0.21 0.87 ± 0.11 0.91 ± 0.11 1.50 ± 0.20 0.31 ± 0.11 0.68 ± 0.48
 2 0.05 ± 0.12 1.50 ± 0.26 0.61 ± 0.10 0.71 ± 0.12 0.39 ± 0.11 0.64 ± 0.16 0.96 ± 0.15 1.87 ± 0.51 1.06 ± 0.27 1.39 ± 0.07 1.12 ± 0.07 2.01 ± 0.27 0.41 ± 0.17 0.98 ± 0.57
 4 0.82 ± 0.08 2.81 ± 0.07 1.63 ± 0.07 2.23 ± 0.20 1.37 ± 0.03 1.97 ± 0.28 2.41 ± 0.23 3.15 ± 0.34 2.40 ± 0.27 2.93 ± 0.28 2.49 ± 0.53 2.96 ± 0.16 1.37 ± 0.14 2.20 ± 0.69
 6 1.63 ± 0.13 3.77 ± 0.17 2.51 ± 0.05 3.40 ± 0.30 1.85 ± 0.11 2.93 ± 0.13 3.66 ± 0.21 4.69 ± 0.06 3.10 ± 0.29 3.63 ± 0.43 3.53 ± 0.13 4.30 ± 0.16 2.34 ± 0.05 3.18 ± 0.87
